# Solid-State Preparation and Characterization of 2-Hydroxypropylcyclodextrins-Iodine Complexes as Stable Iodophors

**DOI:** 10.3390/biom13030474

**Published:** 2023-03-03

**Authors:** Sandro Dattilo, Fabiola Spitaleri, Danilo Aleo, Maria Grazia Saita, Angela Patti

**Affiliations:** 1 CNR-Istituto per i Polimeri, Compositi e Biomateriali, Via Paolo Gaifami 18, I-95126 Catania, Italy; 2 MEDIVIS-Via Carnazza 34 C, I-95030 Catania, Italy; 3 CNR-Istituto di Chimica Biomolecolare, Via Paolo Gaifami 18, I-95126 Catania, Italy

**Keywords:** cyclodextrins, iodine, solid iodophors, UV validated method

## Abstract

The use of iodine as antiseptic poses some issues related to its low water solubility and high volatility. Stable solid iodine-containing formulations are highly advisable and currently limited to the povidone-iodine complex. In this study, complexes of molecular iodine with 2-hydroxypropyl α-, β- and γ-cyclodextrins were considered water-soluble iodophors and prepared in a solid state by using three different methods (liquid-assisted grinding, co-evaporation and sealed heating). The obtained solids were evaluated for their iodine content and stability over time in different conditions using a fully validated UV method. The assessment of the actual formation of an inclusion complex in a solid state was carried out by thermal analysis, and the presence of iodine was further confirmed by SEM/EDX and XPS analyses. High levels of iodine content (8.3–10.8%) were obtained with all the tested cyclodextrins, and some influence was exerted by the employed preparation method. Potential use as solid iodophors can be envisaged for these iodine complexes, among which those with 2-hydroxypropyl-α-cyclodextrin were found the most stable, regardless of the preparation technique. The three prepared cyclodextrin–iodine complexes proved effective as bactericides against *S. epidermidis*.

## 1. Introduction

The use of iodine as an antiseptic has been known since the 19th century. It is still employed as a disinfectant of wounds, skin and surfaces for its low toxicity and a valuable broad spectrum of activity against Gram-positive and Gram-negative bacteria, fungi, protozoa and viruses without a tendency to promote the development of resistant strains [1,2,3,4]. Indeed, molecular I_2_ can freely enter cells and eliminate microorganisms in a non-specific manner, altering electron transport, inhibiting cellular respiration and protein synthesis, destabilizing membranes and denaturing nucleic acids [5,6,7]. However, iodine is sparingly soluble in water and sublimes at room temperature into irritating and bad smell vapors, limiting the preparation of iodine-based stable aqueous formulations. The first attempts to address the poor water-solubility led to the development of formulations containing ethanol up to 70% (iodine tincture), which, although they delivered an increased concentration of elemental iodine compared to aqueous solutions, posed some toxicity and tolerance issues for the high alcohol concentration. Aqueous solutions with a nominal concentration of iodine up to 10% (*w*/*w*) were also obtained by the addition of potassium iodide to shift the formation equilibrium of the more soluble triiodide (I_3_^−^) ion (Lugol’s iodine), but side effects and staining drawbacks are known even for this formulation.

A major breakthrough in the field was the complexation of iodine with soluble organic polymers, from which free molecular iodine could be released in aqueous systems. Among these iodine-releasing agents, also called “iodophors”, the polyvinylpyrrolidone-iodine complex (PVP-I) is nowadays the most commercially important, and it is available in different forms for topical applications [8,9]. PVP-I offers the advantage of being an easy-handling powder with a stable 10% iodine content, delivering free molecular iodine in the 2–20 ppm range [10] on dissolution with an improved safety profile. In the course of a large investigation on the toxicity of PVP-I [8], allergic and contact dermatitis, associated with the carrier povidone rather than the iodine, have been reported as adverse effects [11,12]. Furthermore, inhibitory effects on the growth of human skin fibroblasts [13] and some impairing of the wound healing process have been observed, making PVP-I unsuitable for contact with subcutaneous tissues in deep wounds [14].

In the search for novel iodophors, a variety of polymers [15,16], polymeric nanoparticles [17,18,19], metal–organic frameworks [20,21,22], inorganic composites [23] and hydrogels [24,25] have been considered as carriers for iodine while less attention has been devoted to complexes with small molecules, which could offer different release mechanisms with respect to polymeric delivery systems [26,27,28].

Cyclodextrins (α-, β- and γ-CD) are toroidally-shaped cyclic oligomers formed by six to eight α-D-glucopyranose units, respectively, bound via 1–4 glycosidic linkage and featured by a lipophilic central cavity and a hydrophilic outer surface. They are widely recognized as non-toxic and pharmacologically inactive excipients for drugs. In most cases, they are used as complexing agents to increase the aqueous solubility of poorly soluble drugs and/or improve their bioavailability and stability [29,30,31,32]. Natural and modified CDs have been investigated in solutions for iodine entrapment [33,34,35,36] and environmental applications [37,38]. For most of the inclusion complexes, enhanced solubility, together with decreased release of iodine, have been reported. In a comparative study on iodine complexes with α-CD, β-CD and 2-hydroxypropyl-α-cyclodextrin (HP-α-CD), the same bactericidal activity was observed for all three complexes, but a higher stability constant was determined for the complex with HP-α-CD, which in addition provided more than one hundred-fold increased aqueous concentration of iodine compared to the native CDs [34].

Despite the development of solid iodophors, it is strongly advised for practical use in clinical fields due to the advantages in handling, storage and safety; only a few examples of solid complexes of iodine and CDs are known. Iodine complexes of α-CD [39] and β-CD [40] were prepared by the co-precipitation method and characterized in their solid state by calorimetric measurements and scanning electronic microscopy for surface morphology [41]. In addition, a patented procedure involving precipitation at 0 °C from a hydroalcoholic solution with Tween 80 and potassium iodide additives was developed for the preparation of an iodine inclusion complex with HP-α-CD [42].

**Scheme 1:**
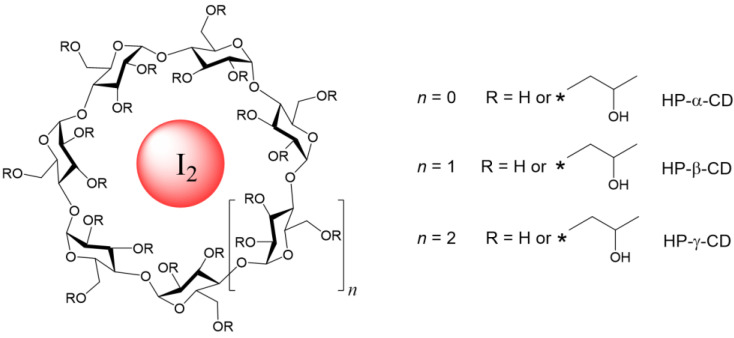
Schematic representation of the investigated cyclodextrin/iodine complexes.

Besides precipitation from solutions of cyclodextrin and a suitable guest molecule, various techniques have been proposed for preparing cyclodextrin inclusion complexes in the solid state [43,44,45,46]. We, therefore, planned to apply some of them to the preparation of cyclodextrin-iodine complexes, focusing our attention on water-soluble 2-hydroxypropyl-cyclodextrins, whose complexes are difficult to obtain in good yield by co-precipitation from aqueous solutions. The study included 2-hydroxypropyl-α- (HP-α-CD), 2-hydroxypropyl-β- (HP-β-CD) and 2-hydroxypropyl-γ- (HP-γ-CD) cyclodextrins (Scheme 1) to evaluate the influence of the preparation method and/or cyclodextrin size on the effectiveness of iodine complexation.

While some investigation has previously been carried out on HP-α-CD/I_2_ complex in solution [34], the corresponding HP-β-CD and HP-γ-CD complexes have not been reported yet, and solid-state characterization has been limited only to iodine complexes with native α- and β-CD [39,40,41].

Complexes of iodine with HP-α-CD, HP-β-CD and HP-γ-CD were prepared using three different solid-state techniques (liquid-assisted grinding, co-evaporation and sealed heating) and characterized by thermal analyses. In addition, scanning electron microscopy coupled with energy-dispersive X-ray analysis (SEM/EDX) was carried out. Here we report the obtained results together with some data on the stability of the obtained complexes in different conditions, targeted to assess their potential as solid iodophors.

## 2. Materials and Methods

### 2.1. Materials

Iodine (sublimated) was from Merck EMSURE^®^ (ACS, ISO, Reag. Ph. Eur.); HP-α-CD (substitution grade, DS 4.8; uma 1252) was purchased from Cyclolab Ltd.(Budapest, Hungary); HP-β-CD (DS 6.9, uma 1537) was from Roquette; HP-γ-CD (DS 4.8, uma 1576) was from Wacker Chemie (Munich, Germany). PVP-I (available iodine 11.0%, dried substance) was purchased by Farmalabor Srl (Canosa di Puglia, Italy). Potassium iodide and 95% EtOH (Eur. Ph.) were purchased from ChemLab NV (Zedelgem, Belgium) and VWR (Milan, Italy), respectively. *Staphylococcus epidermidis* (ATCC^®^ 12228, Origin Strain No. CECT^®^ 231) and all other reagents for the biological assays were obtained from Merck KGaA (Darmstadt, Germany). Bacteria cultures were prepared at 37 °C in 1.5% agar plates supplemented with nutrient broth containing 3% beef extract and 5% peptone.

UV measurements were carried out on an Agilent (Santa Clara, CA, USA) Cary-60 UV-Vis double beam spectrophotometer with 1.5 nm resolution, using polystyrene cuvettes (10 × 10 × 45 mm). Constant temperature at 25 °C was maintained during the analyses.

For the determination of iodine in the samples, to the iodine–CD solid (25 mg) ultrapure water up to 10 g total weight was added, and the solution was stirred for 10 min in a screw cap vial; a known amount (200 mg) of this solution was then diluted with 4.7 g of 1% potassium iodide solution, and the absorbance read at λ = 352 nm. The analyses were carried out in triplicate, and quantification of iodine was performed by external standard (I_3_^−^) calibration.

Stock solution of I_3_^−^ was prepared by dissolving I_2_ (0.591 mM) and KI in 1:1000 molar ratio in ultrapure water and appropriately diluted to build the calibration curve in the concentration range 0.0045–0.0165 mg/g.

The UV-Vis spectrophotometric method was validated (see Supporting Information) with respect to specificity, linearity (R, correlation coefficient = 0.999), accuracy (recovery 99.2 ± 0.44%) and precision (Repeatability: 0.53%; Intermediate precision: 0.75%). No interference due to the presence of the cyclodextrins was observed.

### 2.2. Solid-State Preparation of Iodine–Cyclodextrin Complexes

Physical mixtures (PM) of iodine and cyclodextrins were obtained by 1 min tumble mixing equimolar amounts of the pure components and immediately analyzed.

For the preparation of iodine–cyclodextrin complexes, routine methods were applied as follows:

(a) Liquid-assisted grinding (LAG): In a representative procedure, HP-β-CD (2.50 g, 1.62 mmol) and iodine (412 mg, 1.62 mmol) were mixed in a mortar and EtOH (3 mL) was added. The resultant mixture was kneaded thoroughly with a pestle to obtain homogeneous slurry, and the mixing continued until the solvent was completely removed. The sample was then kept overnight at 25 °C in a ventilated hood, and the resultant dark red solid was finely pulverized.
(b) Co-evaporation (COE): Typically, a solution of HP-β-CD (1.07 g, 0.70 mmol) and iodine (177 mg, 0.70 mmol) in EtOH (3 mL) was taken to dryness under vacuum at 30 °C in a Buchi rotavapor. The obtained dark red solid was then transferred to a petri dish and left overnight at 25 °C in a ventilated hood before the analysis.
(c) Sealed-heating (SH): In a representative example, a physical mixture of HP-β-CD (2.00 g, 1.30 mmol) and iodine (330 mg, 1.30 mmol) was put in a vial crimped with a Teflon cap and maintained at 60 °C in a laboratory oven for 6 h. Then, the vial was allowed to cool to room temperature, and the dark red solid was transferred to a petri dish, left overnight at 25 °C in a ventilated hood, and then analyzed.

### 2.3. Solid-State Characterization of Iodine–Cyclodextrin Complexes

Thermogravimetric analyses (TGA) were performed using a thermogravimetric apparatus (TA Instruments Q500, New Castle, DE, USA) under a nitrogen atmosphere at 10 °C/min heating rate from 50 °C to 800 °C. Sample weights were in the range of 3–6 mg. The weight loss percent and its derivate (DTG) were recorded as a function of temperature.

Fourier Transform Infrared Spectroscopy (FT-IR) of cyclodextrin complex was recorded using a Perkin Elmer Spectrum 100 UATR (Waltham, MA, USA) spectrometer in attenuated total reflectance (ATR) mode. The absorption bands were recorded in 4000 to 600 cm^−1^ with 16 scans and a resolution of 4 cm^−1^. The data were analyzed using OMNIC software.

Scanning electron microscopy/energy-dispersive X-ray analyses (SEM/EDX) were carried out on a Thermo Phenom Prox (Thermo Fisher Scientific, Waltham, MA USA) desktop scanning electron microscopy with a CeB6 source combined with a fully integrated energy-dispersive X-ray (EDX) detector (Silicon Drift Detector). The samples were metalized with a thin layer of gold (10 nm thickness) and subjected to SEM and EDX analysis using an acceleration voltage of 15 kV and a magnification of 2000×.

X-ray photoelectron spectra (XPS) were carried out on a PHI 5000 VersaProbe II (ULVAC-PHI.Inc, Hagisono, Japan) instrument equipped with a monochromatic Al Kα (hν = 1486.6 eV) excitation source. The binding energies were referenced to the C 1*s* peak at 285.0 eV of the surface adventitious carbon.

### 2.4. Stability Studies

(a) Accelerated stability test: Samples of solid complexes (about 1 g each, analyses in triplicate) were spread in an open glass Petri dish (40 mm diameter) and stored at 40 °C in oven for up to 28 days. Aliquots of each sample (25 mg) were withdrawn at set intervals and analyzed for the iodine content by UV.
(b) Real-time stability: The solid complexes obtained by the suitable procedure (1 g each) were stored in a transparent low-density (60 μm thickness) heat-sealed polyethylene bag and kept in the dark at 25 ± 2 °C (65 ± 5% RH). At regular intervals over three months, aliquots (25 mg) of the samples were monitored (analyses in triplicate) for the iodine content by UV.
(c) Stability in solution: Solutions of the solid complexes were prepared in water at a 2.5 mg/mL concentration and sterilized by filtration (CA, 0.2 µm). The solutions were then dispensed in white opaque polyethylene bottles (5 mL) in sterile conditions and kept at room temperature (25 °C ± 2 °C; 65 ± 5% RH) for up to three months. At regular intervals, aliquots of the solution (200 mg) were diluted with 4.7 g of 1% potassium iodide solution and analyzed in triplicate for the iodine content.

### 2.5. Time-Kill Assay

Bacterial suspensions were prepared from 16 h growth cultures diluted to ~1.5 × 10^8^ CFU/mL, as estimated by UV analysis compared to a 0.5 McFarland turbidity standard. The final assay concentration of 6 × 10^5^ CFU/mL of *Staphylococcus epidermidis* was achieved through intermediate bacterial suspensions of ~7.5 × 10^6^ CFU/mL. Subsequently, 0.1 mL of bacterial suspension was added to 1.9 mL of each sample solution and incubated for different times (10, 20, and 40 s, and 1, 2, 4, and 8 min, 1 and 6 h) before residual iodine neutralization with a 0.5% sodium thiosulfate solution (1:10 dilution, *v*/*v*). HP-α-CD/I_2_, HP-β-CD/I_2_ and HP-γ-CD/I_2_ in water (0.025% available iodine concentration) were tested in parallel with blank control and a PVP-I solution (0.025% available iodine concentration) as reference iodophor. After two additional rounds of dilutions (1:10, *v*/*v*) in sterile saline solutions, 0.1 mL of each sample was plated (by spreading) onto an agar-enriched culture medium and incubated at 37 °C (24 h) before colony counting. Results are means ± SEM of three independent experiments performed in duplicate. Statistical analyses were performed with two-way ANOVA, and *p*-values were considered significant at α ≤ 0.05.

## 3. Results and Discussion

### 3.1. Solid-State Preparation of the Iodine–CD Complexes

At the onset of our work, iodine and HP-β-CD were mixed in 1:1 molar ratio, as this stoichiometry has been reported with most iodine–cyclodextrins systems [41]. The preparation of the complex was carried out in parallel by (a) LAG with 95% ethanol as solvent, (b) COE from ethanol, and (c) SH in a sealed vessel at 60 °C for 6 h.

The iodine content in the solids was determined by UV providing that all molecular iodine was converted into triiodide anion (I_3_^−^) by adding a large excess of potassium iodide. A reported method [47] was fully validated for linearity, precision and accuracy (validation parameters in Table S1); this method determined iodine content of 10.9% for a control sample of commercial PVP-I with 11.0% declared title.

Assuming all the determined iodine is hosted in cyclodextrin, at the first approximation, the loading (L) was estimated by the ratio of measured % I_2_ (*w*/*w*) in the sample to the theoretical % I_2_ (*w*/*w*) expected for complete inclusion of the starting amount of iodine, L = [(%I_2sample_/%I_2theor_) × 100].

Complexes prepared with the LAG and COE methods showed comparable levels of iodine loading (Table 1, entries 1–3). In contrast, the SH method appeared more effective, affording a solid with content of iodine quite close to that of PVP-I. Prolonging the heating up to 12 h in the SH method led to only a slight increase in the iodine loading. Conversely, decreasing temperature in the SH method was detrimental (Table 1, compare entries 3 and 5), and even at longer times, rather low levels of iodine in the solid were measured. Increasing the temperature to 80 °C was not applicable since the solid mixture showed a “pseudo”-melted appearance, and extensive sublimation of iodine was observed in the sealed vial.

Higher HP-β-CD:I_2_ molar ratios (2:1 and 4:1) in the SH method at 60 °C led to a similar iodine loading in the obtained solids (Table 1, compare entries 3 with 6 and 7), not improved with respect to that obtained in stoichiometric conditions.

The three protocols for the preparation of iodine complexes were then applied to HP-α-CD and HP-γ-CD. With both these cyclodextrins, the higher iodine loading was again obtained with the SH method (Table 1, entries 8–10 and 11–13). However, good levels of iodine loading were observed in the complexes with HP-α-CD also prepared with LAG or COE procedures (Table 1, compare entry 8 with 1 and 11, and entry 9 with 2 and 12).

While the iodine content in complexes with HP-β-CD and HP-γ-CD was found to be dependent on the preparation method used, in the order LAG < COE < SH, less variability was observed for complexes with HP-α-CD. From the obtained data, the inclusion of iodine in HP-α-CD appeared more favored with respect to the β- and γ-analogs, as yet observed for the inclusion complexes with native cyclodextrins whose formation constants follow the order α > β > γ [33].

### 3.2. Solid-State Characterization of Iodine–CD Complexes

In order to confirm the formation of solid complexes between a given guest and cyclodextrins, different techniques can be applied, and differences in the features of the host–guest interaction mixture with respect to pure components and their physical mixture (PM) are usually considered as a proof of complexation [48].

By applying this approach, the solids obtained were first characterized by thermogravimetric analysis (TGA), then SEM/EDX and XPS techniques were considered to confirm the presence of iodine. Unfortunately, the analysis of IR spectra, which has been shown valuable in confirming complexation in the solid state, was not applicable in our case since iodine is not IR-responsive, and changes in the cyclodextrin features upon complexation were not found easily detectable (Figure S1).

#### 3.2.1. Thermogravimetric Analysis

Data from TGA and relative derivate analyses are summarized in Table 2, in which the maximum of main peaks in the derivative analysis (T*_d_*) and the temperature corresponding to 50% of degradation of the complexes (T*_Δm50%_*) are reported.

**Figure 1:**
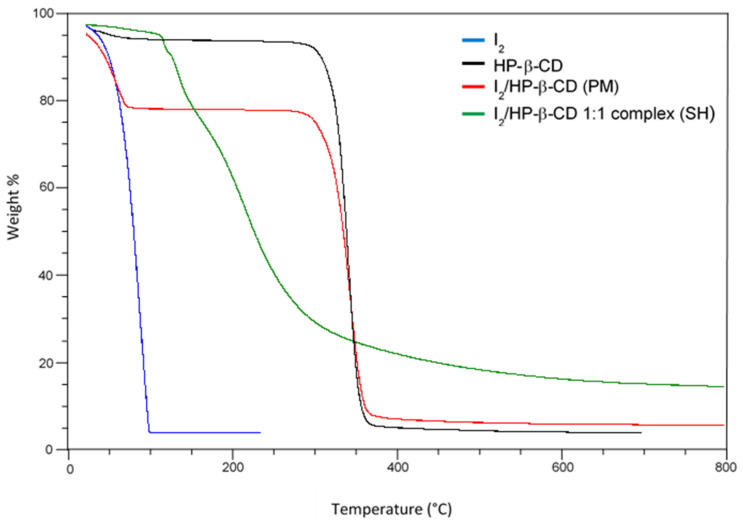
Thermogravimetric (TG) curves of iodine and HP-β-CD as pure components, their 1:1 physical mixture (PM), and 1:1 complex prepared by SH method.

Figure 1 shows the TG curve of the HP-β-CD/I_2_ complex prepared by the SH method compared to the curves of pure components and their equimolar PM. The curve of HP-β-CD showed some weight loss around 100 °C associated with its dehydration, followed by main degradation with a T*_Δm50%_* at 342 °C. Weight losses associated with sublimation/fusion of iodine were observed in the 45–114 °C. While the thermogram of PM appears as a sum of the curves of the pure components, substantial differences can be evidenced in the curve of the complex.

In analogy with the TG data reported for the iodine complex of native β-CD [40], the curve of HP-β-CD/I_2_ complex can be roughly divided into three regions, the first of which, in the range, 45–110 °C, is attributed to the release of some adsorbed iodine and a small amount of water from HP-β-CD.

The second weight loss, occurring in the range of 110–175 °C and better visible as two separate transitions in DTG curves (T*_d1_* and T*_d2_* in Table 2), is attributed to the release of the iodine from HP-β-CD, probably caused by a collapse of the crystal lattice of the complex. Following its interaction with HP-β-CD, the iodine is protected from subliming early and displays higher thermostability than pure iodine. The total weight loss of these two stages accounts for 15% and approximatively matches the weight percentage of iodine determined by UV analysis of the HP-β-CD/I_2_ powder.

Further weight loss at temperatures over 175 °C is attributed to the early degradation of the HP-β-CD molecule in the complex, probably related to a decreased structural stability of the cyclodextrin following the inclusion of iodine. Furthermore, the occurrence of some acid-catalyzed hydrolysis [49], promoted by oxyacids formed by iodine and water at high temperatures, could contribute to the lowering in the degradation temperature of the cyclodextrin in the complex compared to when it is pure. In addition, an increased percentage of residue in the complex with respect to the separate components was detected.

The same thermal features were observed for complexes with HP-α-CD and HP-γ-CD compared to the corresponding physical mixtures and pure components (Figures S2 and S3).

**Figure 2:**
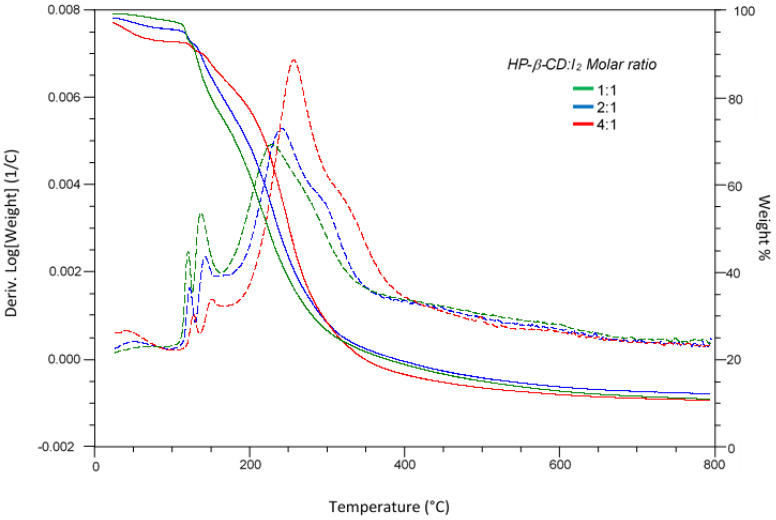
TG (solid lines) and first derivative (dotted lines) curves of HP-β-CD/I_2_ complexes prepared by SH method in 1:1, 2:1 and 4:1 HP-β-CD:I_2_ molar ratios.

Figure 2 shows the thermograms of HP-β-CD/I2 complexes prepared by the SH method using different HP-β-CD to I2 ratios. Increasing the host:guest ratio resulted in a progressive increase in the degradation temperature of cyclodextrin (compare entries 6–8 for T*_d3_* in Table 2), while a shoulder at higher temperatures (T*_d4_* in Table 2) became visible in the TG curves. This additional transition could be attributed to some uncomplexed cyclodextrin, which is, however, affected by the presence of iodine and still undergoes early decomposition compared to the pure molecule.

**Figure 3:**
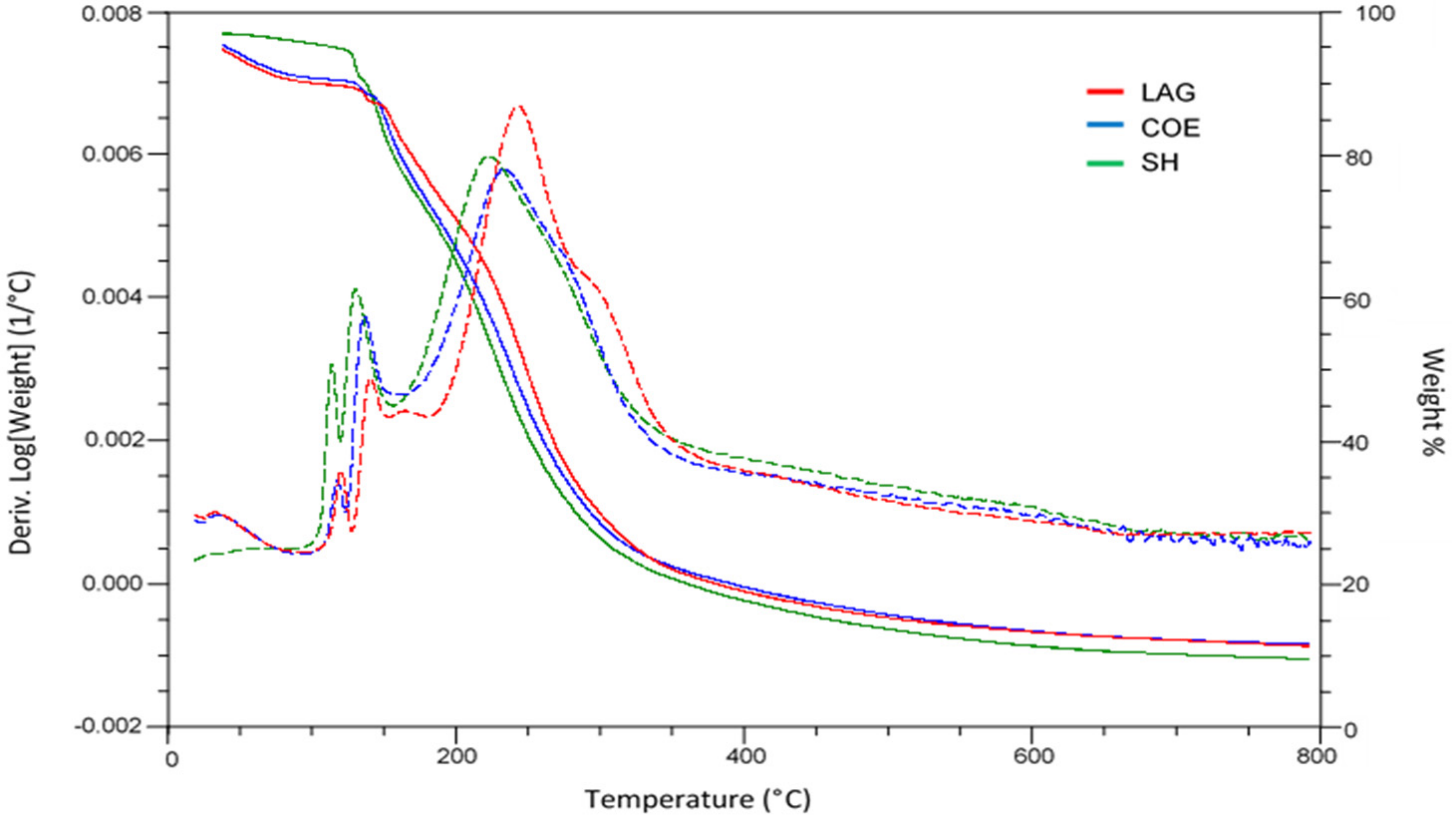
TG (solid lines) and first derivative (dotted lines) curves of iodine/HP-β-CD complexes prepared by LAG, COE and SH methods.

Figure 3 compares the TG curves for the 1:1 complexes of iodine with HP-β-CD prepared with the three different methods. The transition related to uncomplexed cyclodextrin (T*_d4_* in Table 2) was not evident for the SH and COE samples, while it was more visible for the LAG sample (compare entries 4–6 in Table 2). Independently by the employed preparation method, this transition was instead present in the TG curves of complexes with HP-γ-CD and absent for HP-α-CD, as can be better appreciated from the DTG curves at temperatures around 300 °C (Figures S4 and S5).

From these data, it could be suggested that the inclusion of iodine best fits with the smaller size of the cavity in HP-α-CD, and the efficiency of complexation seems to be affected by the preparation method mainly for the HP-β-CD/I_2_ solid.

#### 3.2.2. Scanning Electron Microscopy/Energy-Dispersive X-ray Analysis (SEM/EDX)

SEM coupled with EDX analysis was applied to iodine complexes HP-α-CD, HP-β-CD and HP-γ-CD prepared by the SH method to evaluate their size, morphology and elemental composition.

**Figure 4:**
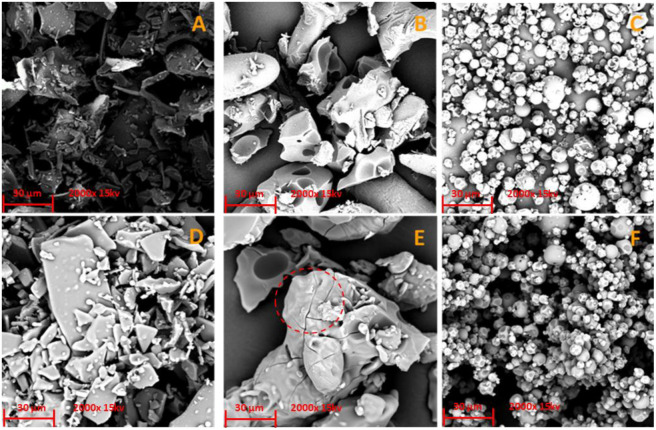
SEM images (2000×) of pure HP-α-CD (**A**), HP-β-CD (**B**) and HP-γ-CD (**C**) and the corresponding HP-α-CD/I_2_ (**D**), HP-β-CD/I_2_ (**E**) and HP-γ-CD/I_2_ (**F**) prepared by SH method.

Compared to pure HP-α-CD (Figure 4A), the corresponding HP-α-CD/I_2_ (Figure 4D) sample showed the presence of some smaller rounded structures. The particles of HP-β-CD/I_2_ solid showed greater roughness with respect to HP-β-CD (Figure 4, compare B,E), while no substantial changes were evidenced going from HP-γ-CD to the corresponding complex HP-γ-CD/I_2_ (Figure 4, compare C,F), probably due to the much smaller particle size of this cyclodextrin.

The amount of iodine in the samples, as an average of three measurements over areas of approx. 400 µm^2^ was measured using the EDX analyzer, and the results are reported in Table 3. Although the iodine content determined by this method was lower than that measured by UV, the obtained values are comparable to those obtained from the accelerated stability study (40 °C, 28 days) for samples prepared by SH procedure (see *infra*). Since the metallization process required to prepare the samples for SEM analyses is carried out under high vacuum, a loss of iodine from cyclodextrin to an extent comparable to that occurring by prolonged heating may be suggested.

Apart from the chemical nature of the cyclodextrin host, also the solid-state preparation could account for the different morphology observed for our complexes and that reported for α-CD/I_2_ and β-CD/I_2_ complexes [41], obtained as needle-like crystals of regular size by precipitation from solutions containing iodine and the cyclodextrin host.

#### 3.2.3. X-ray Photoelectron Spectroscopy (XPS)

**Figure 5:**
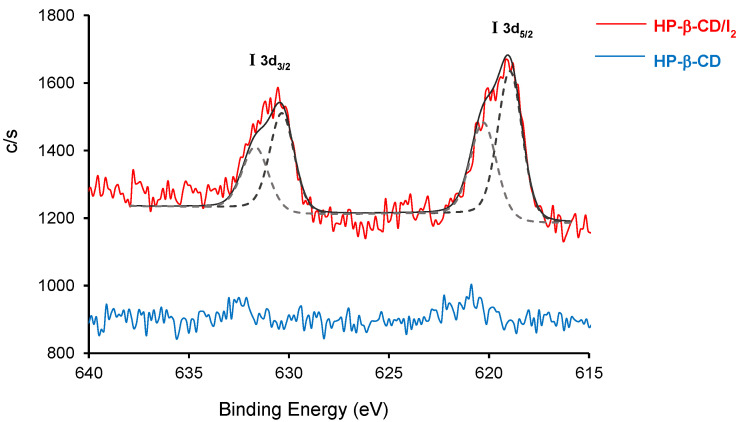
Iodine region of XPS spectra for HP-β-CD/I_2_ and HP-β-CD solids.

The XPS survey profile of HP-β-CD/I_2_ solids confirms the presence of I, C and O (Figure S5) and two characteristic peaks for I 3*d*_3/2_ and I 3*d*_5/2_ were observed. Deconvolution of these peaks into two more intense peaks at 618.9 and 630.2 eV, ascribed to I_3_^−^, and two smaller shoulder peaks at 620.2 and 631.8 eV, corresponding to I_2_, suggested the presence of I_2_ in different states (Figure 5) [50] resulting by the partial transformation of I_2_ into I_3_^−^ after interaction with the cyclodextrin host. XPS spectra of HP-α-CD/I_2_ and HP-γ-CD/I_2_ showed the same features without significant changes in binding energies of the observed peaks compared to the HP-β-CD/I_2_ complex.

### 3.3. Stability Studies

**Figure 6:**
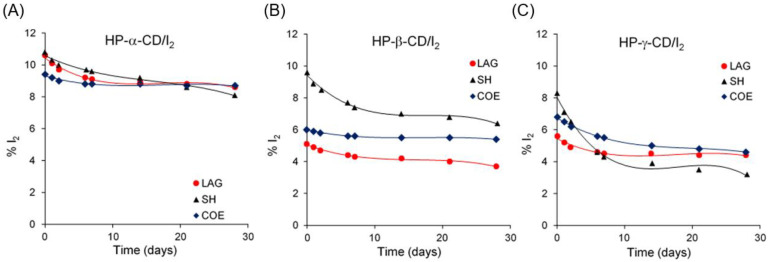
Variation of the iodine content with time in the accelerated stability testing of (**A**) HP-α-CD/I_2_, (**B**) HP-β-CD/I_2_ and (**C**) HP-γ-CD/I_2_ complexes prepared by LAG, COE or SH method.

In order to evaluate the long-term stability of the obtained iodine/CD complexes, all the prepared solids were subjected to accelerated stability tests by spreading the samples on a Petri dish kept at 40 °C in a ventilated hood and monitored for their iodine content over 28 days. The obtained results, reported in Table S2 and plotted in Figure 6, evidenced a similar trend in the global decrease (18–33% of the initial values) of iodine content for all the samples, except for the HP-γ-CD/I_2_ complex prepared by the SH method, that retained only 39% of the initial iodine after 28 days. Most of the iodine loss was observed in the first seven days, with marked differences between the three complexes also depending on the preparation method; then, the concentration of iodine remained almost constant.

For HP-α-CD/I_2,_ similar stability profiles were observed for the complexes prepared with the different methods. Still, in the first seven days, the LAG sample showed a greater decrease in the iodine content compared to COE and SH samples, for which the loss of iodine occurred more gradually. At the end of the experiment, the samples prepared with LAG and COE methods converged to the same iodine content, which was slightly higher than that retained in the SH solid (Figure 6A).

Comparable global loss of iodine (27% vs. 33%) with respect to the initial content was observed for LAG HP-β-CD/I_2_ and SH HP-β-CD/I_2_ samples over 28 days, while the COE solid displayed a lower decrease (10%). Unlike what was observed with HP-α-CD, the differences in the initial iodine loading in the complexes were roughly maintained, and the final iodine content in the three samples did not converge toward a common value (Figure 6B). The LAG and COE samples of HP-γ-CD/I_2_, albeit with different kinetics, reached the same level of iodine content at the end of the experiment, in analogy with what was observed with HP-α-CD. The SH preparation of HP-γ-CD/I_2_ underwent an approximately 50% decrease in its initial iodine content in just seven days, retaining the lowest amount of iodine of all the tested samples (Figure 6C).

From these data, it can be deduced that all the complexes could contain some iodine interacting with the external surface of the cyclodextrin, in addition to that included in the cavity, and for this reason, more sensitive to being lost first during the test. For all three tested cyclodextrins, the LAG and COE preparations showed lower long-term variability in their iodine content than the corresponding SH samples, whose iodine decrease was observed continuously over time. However, in the case of the HP-β-CD/I_2_ complex, the higher initial iodine loading of the SH preparation compared to the LAG and COE samples compensates for its tendency to lose iodine over time, allowing it to maintain a higher iodine level than the complexes prepared with the other two methods throughout the experiment.

Real-time stability of HP-α-CD/I_2_ (LAG preparation), HP-β-CD/I_2_ (SH preparation) and HP-γ-CD/I_2_ (COE preparation), which gave the best result in the accelerated stability test, was then evaluated by keeping the samples in a closed low-density polyethylene bag at 25 °C, as the worst storage conditions. The iodine content was monitored over 3 months compared to solid PVP-I, taken as a reference. Pleasantly, the HP-α-CD/I_2_ complex showed a nearly unchanged iodine content after 3 months, proving to be stable as PVP-I. Instead, at the same time, approximately 15% of the starting iodine was lost from HP-β-CD/I_2_ and HP-γ-CD/I_2_ samples (Figure S7).

While most of the previously reported cyclodextrin–iodine complexes have been investigated in aqueous solution, wherein equilibria between iodine and polyiodide anions are known to occur and are directly affected by interactions with water, it is the first time that the stability over time of such complexes is evaluated on solids.

All the obtained complexes are fully soluble in water in the range of concentration usually applied for pharmaceutical formulations (1–10% *w*/*w*), and the assessment of their stability in solution deserves focused investigation. However, since it is known that the use of dilute solutions of PVP-I, which show higher antibacterial activity compared to the more concentrated ones [51], is strongly limited by their low storage stability [52], a preliminary test was carried out with 0.25% (*w*/*w*) solutions of iodine/CD complexes to evaluate their potential as alternative iodophors in this critical concentration. Unfortunately, already after two months at 25 °C in low-density polyethylene bottles, all solutions, including that of PVP-I, showed a complete loss of iodine, with the interesting exception of those containing the HP-α-CD/I_2_ complexes, which still retained about 50% of the initial iodine content (Table S3).

### 3.4. Antibacterial Activity

It has already been reported that some cyclodextrin–iodine complexes, including HP-α-CD/I_2_, display the same bactericidal activity of PVP-I against *Staphylococcus aureus* [34], indicating that the presence of the cyclodextrin host does not affect the biological activity of the delivered iodine. On this basis, it could be expected that the HP-(α,β,γ)-CD/I_2_ complexes described here have biological activities not different from those reported for other iodine-containing formulations such as PVP-I.

The HP-α-CD/I_2,_ HP-β-CD/I_2_ and HP-γ-CD/I_2_ complexes were then evaluated for their antibacterial activity against the PVP-I-sensitive *Staphylococcus epidermidis* strain [53] by time-kill assay. All three complexes, in 0.025% available iodine concentration, gave a 99.99% reduction of the bacterial cell vitality (corresponding to a CFU/mL reduction > 5 Log10) as early as 10 s after the contact time, and the same result was observed with PVP-I used as reference iodophor.

## 4. Conclusions

The solid-state preparation of complexes of iodine with three 2-hydroxypropyl cyclodextrins differing for their size cavity was carried out with three different methods, and the efficiency in the iodine loading was compared. The sealed heating method proved to be the most efficient in providing high initial levels of iodine loading, but the obtained solids suffered some loss of iodine over time, and sufficient long-term stability in complexes prepared by this technique was observed only with 2-hydroxypropyl-β-cyclodextrin. On the other hand, co-evaporation and liquid-assisted grinding procedures are preferable for obtaining long-term, more stable complexes of iodine with the α- or γ-analogs.

Thermal analysis of the obtained solids supported the formation of inclusion complexes with all three tested cyclodextrins and revealed some differences related to the employed preparation method. Accelerated stability tests evidenced that complexes with 2-hydroxypropyl-α-cyclodextrin were the most stable independently by the preparation method and converged toward the same iodine content over time. The same complex also showed unchanged iodine content for up to three months at 25 °C, offering a valuable potential alternative as iodophor to the most popular povidone-iodine, considering the presence of the cyclodextrin host does not affect the antibacterial activity of the delivered iodine.

Further study on the stability of these iodine/CD complexes in solution, especially in the concentration ranges wherein the stability of povidone-iodine is critical and/or in different formulations, is currently underway.

## Figures and Tables

**Table 1:** Content of iodine in solid complexes with cyclodextrins.

Entry	Cyclodextrin	Preparation Method *^a^*	Temperature (°C)	% Iodine (*w*/*w*) *^b^*	% Loading *^c^*
1	HP-β-CD	LAG	25	5.14 ± 0.03	36.2
2	HP-β-CD	COE	30	6.01 ± 0.04	42.2
3	HP-β-CD	SH	60	9.64 ± 0.03	67.9
4	HP-β-CD	SH *^d^*	60	9.78 ± 0.05	68.9
5	HP-β-CD	SH *^d^*	40	4.40 ± 0.04	31.0
6	HP-β-CD	SH *^e^*	60	4.71 ± 0.02	61.8
7	HP-β-CD	SH *^f^*	60	2.40 ± 0.06	60.6
8	HP-α-CD	LAG	25	10.58 ± 0.02	62.7
9	HP-α-CD	COE	30	9.45 ± 0.03	55.9
10	HP-α-CD	SH	60	10.81 ± 0.01	63.9
11	HP-γ-CD	LAG	25	5.56 ± 0.05	40.0
12	HP-γ-CD	COE	30	6.77 ± 0.05	48.7
13	HP-γ-CD	SH	60	8.32 ± 0.03	59.9

*^a^* See experimental section for details; *^b^* Determined by UV; *^c^* Referred to the initial iodine amount; *^d^* Sealed heating prolonged up to 12 h; *^e^* HP-β-CD:I_2_ 2:1 molar ratio; *^f^* HP-β-CD:I_2_ 4:1 molar ratio.

**Table 2:** Thermogravimetric data for iodine–cyclodextrin complexes *^a^*.

Entry	Cyclodextrin	Prep. Method	T*_d1_* (°C) *^b^*	T*_d2_* (°C) *^b^*	T*_d3_* (°C) *^b^*	T*_d4_* (°C) *^b^*	T*_Dm=50%_* (°C) *^c^*	% R *^d^*
1	HP-α-CD	PM	-	-	363	-	341	2.3
2	HP-β-CD	PM	-	-	364	-	342	1.7
3	HP-γ-CD	PM	-	-	342	-	330	2.6
4	HP-β-CD	LAG	136	153	258	303	253	12.9
5	HP-β-CD	COE	136	155	249	-	243	13.2
6	HP-β-CD	SH	128	148	240	-	234	11.1
7	HP-β-CD	SH *^e^*	134	153	250	299	248	12.3
8	HP-β-CD	SH *^f^*	139	161	266	320	261	10.8
9	HP-α-CD	LAG	134	151	226	-	236	19.9
10	HP-α-CD	COE	133	148	230	-	233	15.3
11	HP-α-CD	SH	130	148	241	-	232	13.6
12	HP-γ-CD	LAG	145	160	255	295	253	12.7
13	HP-γ-CD	COE	141	157	250	288	248	13.6
14	HP-γ-CD	SH	139	163	256	300	251	12.5

*^a^* Unless otherwise specified, the complexes were prepared by using iodine and cyclodextrin in 1:1 molar ratio; *^b^* Temperature at the maximum rate of degradation step; *^c^* Temperature of 50% weight loss; *^d^* Residue (%) at 800 °C; *^e^* HP-β-CD:I_2_ 2:1 molar ratio; *^f^* HP-β-CD:I_2_ 4:1 molar ratio.

**Table 3:** Iodine % determination by EDX analysis.

Complex	I%	I% (UV) *^a^*
HP-α-CD/I_2_	7.7 ± 0.3	10.26 ± 0.02
HP-β-CD/I_2_	7.2 ± 0.2	8.75 ± 0.06
HP-γ-CD/I_2_	2.9 ± 0.2	7.62 ± 0.09

*^a^* Determined by UV method before EDX analysis.

## Data Availability

The data presented in this study are contained in this article and its Supplementary Materials.

## Supplementary Materials

The following supporting information can be downloaded at: https://www.mdpi.com/article/10.3390/biom13030474/s1, Table S1. ANOVA one factor analysis in validation of the UV method; Figure S1. IR spectra of HP-β-CD/I2 complex and pure HP-β-CD; Figure S2. TG curves of HP-α-CD/I2 complex and the pure components; Figure S3. TG curves of HP-γ-CD/I2 complex and the pure components; Figure S4. TG curves of HP-α-CD/I2 complexes prepared with different methods; Figure S5. TG curves of HP-γ-CD/I2 complexes prepared with different methods; Table S2. Accelerated stability test on solid iodine–cyclodextrin complexes; Figure S6. XPS survey spectrum of HP-β-CD and HP-β-CD/I2 (SH sample) solids; Figure S7. Variation of iodine content of iodine–cyclodextrin complexes on storage at 25 °C in polyethylene bag; Table S3. Variation of iodine content of iodine–cyclodextrin complexes in solution; Table S4. % Inhibition of *S. epidermis* cell viability.

## References

[1] Vermeulen H., Westerbos S.J., Ubbink D.T. (2010). Benefit and harm of iodine in wound care: A systematic review. J. Hosp. Infect..

[2] Lachapelle J.-M., Castel O., Fueyo Casado A., Leroy B., Micali G., Tennstedt D., Lambert J. (2013). Antiseptics in the era of bacterial resistance: A focus on povidone iodine. Clin. Pract..

[3] Capriotti K., Pelletier J., Barone S., Capriotti J. (2018). Efficacy of dilute povidone-iodine against multi- drug resistant bacterial biofilms, fungal biofilms and fungal spores. Clin. Res. Dermatol..

[4] Anderson D.E., Sivalingam V., Eng A., Kang Z., Ananthanarayanan A., Arumugam H., Jenkins T.M., Hadjiat Y., Eggers M. (2020). Povidone-iodine demonstrates rapid in vitro virucidal activity against SARS-CoV-2, the virus causing COVID-19 disease. Infect. Dis. Ther..

[5] Schreier H., Erdos G., Reimer K., König B., König W., Fleischer W. (1997). Molecular effects of povidone-iodine on relevant microorganisms: An electron-microscopic and biochemical study. Dermatology.

[6] Sriwilaijaroen N., Wilairat P., Hiramatsu H., Takahashi T., Suzuki T., Ito M., Ito Y., Tashiro M., Suzuki Y. (2009). Mechanisms of the action of povidone-iodine against human and avian influenza A viruses: Its effects on hemagglutination and sialidase activities. Virol. J..

[7] Lepelletier D., Maillard J.Y., Pozzetto B., Simone A. (2020). Povidone iodine: Properties, mechanisms of action, and role in infection control and Staphylococcus aureus decolonization. Antimicrob. Agents Chemother..

[8] Bigliardi P.L., Abdul S., Alsagoff L., El-Kafrawi H.Y., Pyon J.-K., Tse C., Wa C., Villa M.A. (2017). Povidone iodine in wound healing: A review of current concepts and practices. Int. J. Surg..

[9] Kurakula M., Rao G.S.N.K. (2020). Pharmaceutical assessment of polyvinylpyrrolidone (PVP): As excipient from conventional to controlled delivery systems with a spotlight on COVID-19 inhibition. J. Drug Deliv. Sci. Technol..

[10] Atemnkeng M.A., Plaizier-Vercammen J., Schuermans A. (2006). Comparison of free and bound iodine and iodide species as a function of the dilution of three commercial povidone–iodine formulations and their microbicidal activity. Int. J. Pharm..

[11] Van Ketel W.G., van den Berg W.H. (1980). Sensitization to Povidone-Iodine. Dermatol. Clin..

[12] Lachapelle J.M. (2005). Allergic contact dermatitis from povidone-iodine: A re-evaluation study. Contact Dermat..

[13] Balin A.K., Pratt L. (2002). Dilute povidone-iodine solutions inhibit human skin fibroblast growth. Dermatol. Surg..

[14] Kramer S.A. (1999). Effect of povidone-iodine on wound healing: A review. J. Vasc. Nurs..

[15] Sibbald R.G., Leaper D.J., Queen D. (2011). Iodine made easy. Wounds Int..

[16] Makhayeva D.N., Irmukhametova G.S., Khutoryanskiy V.V. (2020). Polymeric iodophors: Preparation, properties, and biomedical applications. Rev. J. Chem..

[17] Mallick S., Sharma S., Banerjee M., Ghosh S.S., Chattopadhyay A., Paul A. (2012). Iodine-stabilized Cu nanoparticle chitosan composite for antibacterial applications. ACS Appl. Mater. Interfaces.

[18] Gao T., Fan H., Wang X., Gao Y., Liu W., Chen W., Dong A., Wang Y.-J. (2017). Povidone–iodine-based polymeric nanoparticles for antibacterial applications. ACS Appl. Mater. Interfaces.

[19] Tang C., York A.W., Mikitsh J.L., Wright A.C., Chacko A.-M., Elias D.R., Xu Y., Lim H.-K., Prud’homme R.K. (2018). Preparation of PEGylated iodine-loaded nanoparticles via polymer-directed self-assembly. Macromol. Chem. Phys..

[20] Au-Duong A.-N., Lee C.-K. (2017). Iodine-loaded metal-organic framework as growth-triggered antimicrobial agent. Mater. Sci. Eng. C.

[21] Xie W., Cui D., Zhang S.-R., Xu Y.-H., Jiang D.-L. (2019). Iodine capture in porous organic polymers and metal–organic frameworks materials. Mater. Horiz..

[22] Nakhaei M., Akhbari K., Davoodi A. (2021). Biocompatible MOF-808 as an iodophor antimicrobial agent with controlled and sustained release of iodine. CrystEngComm.

[23] Muhire C., Reda A.T., Zhang D., Xu X., Cui C. (2022). An overview on metal Oxide-based materials for iodine capture and storage. Chem. Eng. J..

[24] Mishra A., Chaudhary N. (2010). Study of povidone iodine loaded hydrogels as wound dressing material. Trends Biomater. Artif. Organs.

[25] Gull N., Khan S.M., Khalid S., Zia S., Islam A., Sabir A., Sultan M., Hussain F., Khan R.U., Butt M.T.Z. (2020). Designing of biocompatible and biodegradable chitosan based crosslinked hydrogel for in vitro release of encapsulated povidone-iodine: A clinical translation. Int. J. Biol. Macromol..

[26] Stella V.J., Rao V.M., Zannou E.A., Zia V. (1999). Mechanisms of drug release from cyclodextrin complexes. Adv. Drug Deliv. Rev..

[27] Root-Bernstein R.S., Dillon P.F. (2008). Small molecule complementarity as a source of novel pharmaceutical agents and combination therapies. Curr. Pharm. Des..

[28] Renfrew A.K. (2014). Transition metal complexes with bioactive ligands: Mechanisms for selective ligand release and applications for drug delivery. Metallomics.

[29] Irie T., Uekama K. (1997). Pharmaceutical applications of cyclodextrins. III. Toxicological issues and safety evaluation. J. Pharm. Sci..

[30] Chaudhary V.B., Patel J.K. (2013). Cyclodextrin inclusion complex to enhance solubility of poorly water soluble drugs: A review. Int. J. Pharm. Sci. Res..

[31] Popielec A., Loftsson T. (2017). Effects of cyclodextrins on the chemical stability of drugs. Int. J. Pharm..

[32] Jacob S., Nair A.B. (2018). Cyclodextrin complexes: Perspective from drug delivery and formulation. Drug Devel. Res..

[33] Sanemasa I., Kobayashi T., Deguchi T. (1985). Formation constants of cyclodextrin inclusion complexes with iodine in aqueous solutions. Bull. Chem. Soc. Jpn..

[34] Tomono K., Goto H., Suzuki T., Ueda H., Nagai T., Watanabe J. (2002). Interaction of iodine with 2-hydroxypropyl-α-cyclodextrin and its bactericidal activity. Drug Devel. Ind. Pharm..

[35] Neoh T.-L., Noda Y., Yoshii H., Furuta T. (2006). Release characteristics of iodine encapsulated in cyclodextrins. J. Inc. Phenom. Macrocycl. Chem..

[36] Asim M.H., Jalil A., Shahzadi I., Khan M., Matuszczak B., Bernkop-Schnürch A. (2019). Mucoadhesive *S*-protected thiolated cyclodextrin-iodine complexes: A promising strategy to prolong mucosal residence time of iodine. Future Microbiol..

[37] Szente L., Fenyvesi É., Szejtli J. (1999). Entrapment of iodine with cyclodextrins: Potential application of cyclodextrins in nuclear waste management. Environ. Sci. Technol..

[38] Hirota M., Higaki S., Ito S., Ishida Y., Terao K. (2021). Effects of 2-hydroxypropyl α-cyclodextrin on the radioactive iodine sorption on activated carbon. J. Radioanal. Nucl. Chem..

[39] Lechat F.L., Wouessidjewe D., Herrenknecht C., Duchěne D. (1992). Preparation and stability of iodine/α-cyclodextrin inclusion complex. Drug Devel. Ind. Pharm..

[40] Wang T., Li B., Feng Y., Guo Q. (2011). Preparation, quantitative analysis and bacteriostasis of solid state iodine inclusion complex with β-cyclodextrin. J. Incl. Phenom. Macrocycl. Chem..

[41] Polumbryk M., Pasichnyi V., Omelchenko C., Vyshnevskiy O. (2017). Determination of structure and morphology of the cyclodextrins-iodine complexes. Ukr. Food J..

[42] Guiming L., Song L.T., Zhao M., Yang Z., Feng S., Huang M., Fu Z., Zhang J., Li W., Qin Y. (2017). High-Stability Cydiodine Powder as Well as Preparation Method and Application Method Thereof. Chinese Patent.

[43] Nakai Y., Yamamoto K., Terada K., Watanabe D. (1987). New methods for preparing cyclodextrin inclusion compounds. I. Heating in a sealed container. Chem. Pharm. Bull..

[44] Kreaz R.M.A., Abu-Eida E.Y., Erős I., Kata M. (1999). Freeze-dried complexes of furosemide with β-cyclodextrin derivatives. J. Incl. Phen. Macroc. Chem..

[45] Al Hagbani T., Nazzal S. (2017). Curcumin complexation with cyclodextrins by the autoclave process: Method development and characterization of complex formation. Int. J. Pharm..

[46] Jug M., Mura P.A. (2018). Grinding as solvent-free green chemistry approach for cyclodextrin inclusion complex preparation in the solid state. Pharmaceutics.

[47] Pursell J.L., Pursell C.J. (2016). Host–guest inclusion complexation of α-cyclodextrin and triiodide examined using UV–vis spectrophotometry. J. Phys. Chem. A.

[48] Mura P. (2015). Analytical techniques for characterization of cyclodextrin complexes in the solid state: A review. J. Pharm. Biomed. Anal..

[49] Szejtli J., Budai Z. (1976). Acid hydrolysis of β-cyclodextrin. Acta Chim. Hung..

[50] Lee Y.R., Do X.H., Cho K.Y., Jeong K., Baek K.-Y. (2020). Amine-functionalized zeolitic imidazolate framework-8 (ZIF-8) nanocrystals for adsorption of radioactive iodine. ACS Appl. Nano Mater..

[51] Berkelman R.L., Holland B.W., Anderson R.L. (1982). Increased bactericidal activity of dilute preparations of povidone-iodine solutions. J. Clin. Microbiol..

[52] Bhagwat D., Iny O., Pedi F. (1991). Stabilizing Packaged Iodophor and Minimizing Leaching of Iodine through Packaging. U.S. Patent.

[53] Musumeci R., Bandello F., Martinelli M., Calaresu E., Cocuzza C.E. (2019). In vitro bactericidal activity of 0.6% povidone-iodine eye drops formulation. Eur. J. Ophthalmol..

