# Supplementary material for: Solid-State Preparation and Characterization of 2-Hydroxypropylcyclodextrins-Iodine Complexes as Stable Iodophors
Source: Biomolecules. 2023 Mar 3;13(3):474. doi: 10.3390/biom13030474 (PMC10046614; doi:10.3390/biom13030474)
Supplement: Supplementary file 1 [file biomolecules-13-00474-s001.zip › biomolecules-2208659-supplementary.pdf]

Solid-State Preparation and Characterization of 2-Hydroxypropylcyclodextrins-Iodine Complexes as Stable Iodophors

Sandro Dattilo [1], Fabiola Spitaleri [2], Danilo Aleo [2,*], Maria Grazia Saita [2] and Angela Patti [3,*]

[1] CNR-Istituto per i Polimeri, Compositi e Biomateriali, Via Paolo Gaifami 18, I-95126 Catania, Italy
[2] MEDIVIS-Via Carnazza 34 C, I-95030 Catania, Italy
[3] CNR-Istituto di Chimica Biomolecolare, Via Paolo Gaifami 18, I-95126 Catania, Italy
* Correspondence: danilo.aleo@medivis.it (D.A.); angela.patti@cnr.it (A.P.)

Validation of the UV method for iodine determination — Page SI-1

Table S1. ANOVA one factor analysis (α=0.05) — SI-2

Figure S1. IR spectra of HP-β-CD and HP-β-CD/I_2 complex — SI-3

Figure S2. TG curves of HP-α-CD/I_2 complex and the pure components — SI-4

Figure S3. TG curves of HP-γ-CD/I_2 complex and the pure components — SI-4

Figure S4.TG curves of HP-α-CD/I_2 complexes prepared with different methods — SI-5

Figure S5. TG curves of HP-γ-CD/I_2 complexes prepared with different methods — SI-5

Figure S6. XPS survey spectrum of HP-β-CD and HP-β-CD/I_2 (SH sample) solids — SI-6

Table S2. Accelerated stability test on solid iodine-cyclodextrin complexes — SI-7

Figure S7. Variation of iodine content of iodine-cyclodextrin complexes on storage at 25 °C in polyethylene bag — SI-7

Table S3. Variation of iodine content of iodine-cyclodextrin complexes in solution — SI-8

Table S4. % Inhibition of *S. epidermis* cell viability — SI-8

Validation of the UV method for iodine determination

Linearity and Range

For linearity study, six solutions having different iodine concentration in the range 0.0045-0.0165 mg/g were analyzed. The obtained data were fitted to the model y = ax + b using least-squares regression and a linear relationship with the equation $y = 102.07x + 0.0196$ ($R^2 = 0.9999$) was found over the considered range.

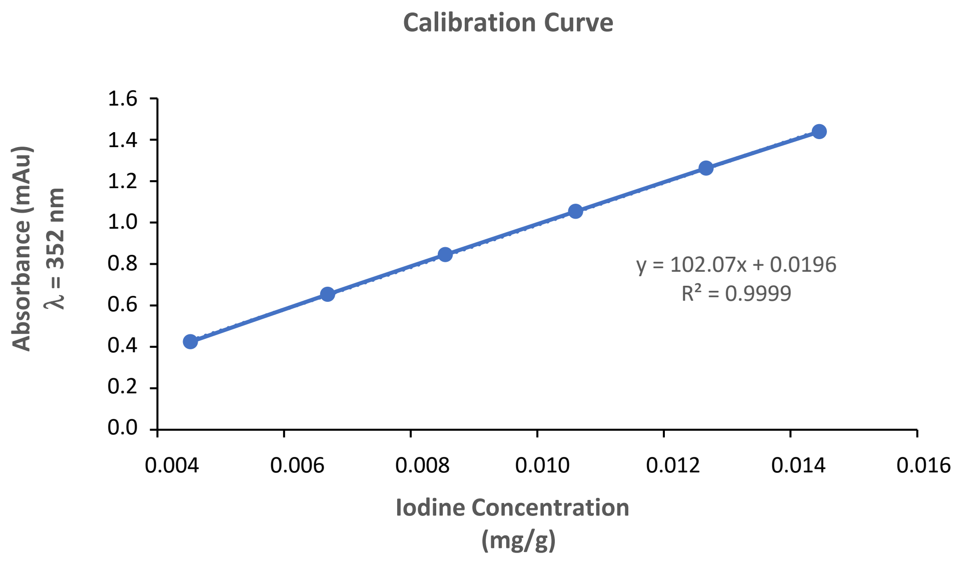

Accuracy

Accuracy of the method was studied by recovery experiments. The recovery was performed at three levels, 0.090, 0.10 and 0.13 mg/g of iodine concentrations using PVP-I as reference standard. Six samples were prepared for each recovery level. The percentage recoveries were calculated from the calibration curve. Accuracy is given as $M \pm \left(\frac{SD}{\sqrt{n}}\right) t$, where M is the overall mean value from recovery testing, SD is the Standard Deviation, and t is the *student's t* (0.05, 18) = 2.101. From the data accuracy of 99.2% ± 0.40% and RSD = 0.80% were determined.

Precision

The precision of the analytical method was obtained as intra-day variation (repeatability) and inter-day variation (intermediate precision) studies.

Intra-day and inter-day data were taken from the recoveries of six independent samples of PVP-I (iodine concentration 0.010%) in the same day and in three different days, respectively.

The coefficient of variation of the repeatability CV_r and the coefficient of variation of the intermediate precision CV_R were determined by one-way analysis of variance (ANOVA) by setting a significance level α=0.05 (Table SI-1).

**Table S1**: ANOVA one factor analysis (α=0.05)		
**Repeatability**	Intra assay variance ($\boldsymbol{S_r^2}$)	0.28
	Standard deviation intra assay ($\boldsymbol{S_r}$)=$\sqrt{}$ $\boldsymbol{S_r^2}$	0.53
	**CV$_r$** repeatability (100 x S_r/M)	0.53%
**Intermediate precision**	Intermediate precision variance ($\boldsymbol{S^2_R}$) = $[(S^2_r)+(S^2_{xm}-S^2_r/n)]^a$	0.54
	Standard deviation Inter assay (S_R)= $\sqrt{}$ $\boldsymbol{S_R^2}$	0.74
	**CV$_R$** Intermediate precision (100 x S_r/M)	0.74%
$^aS^2_{xm}$ = Variance of mean		

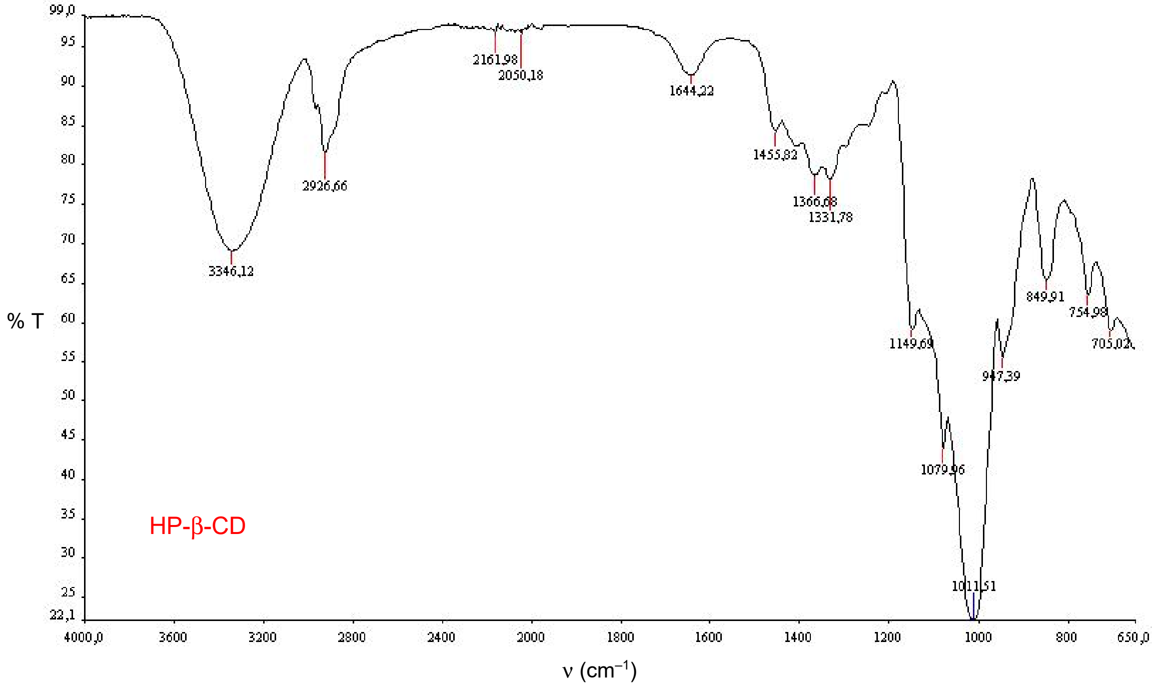

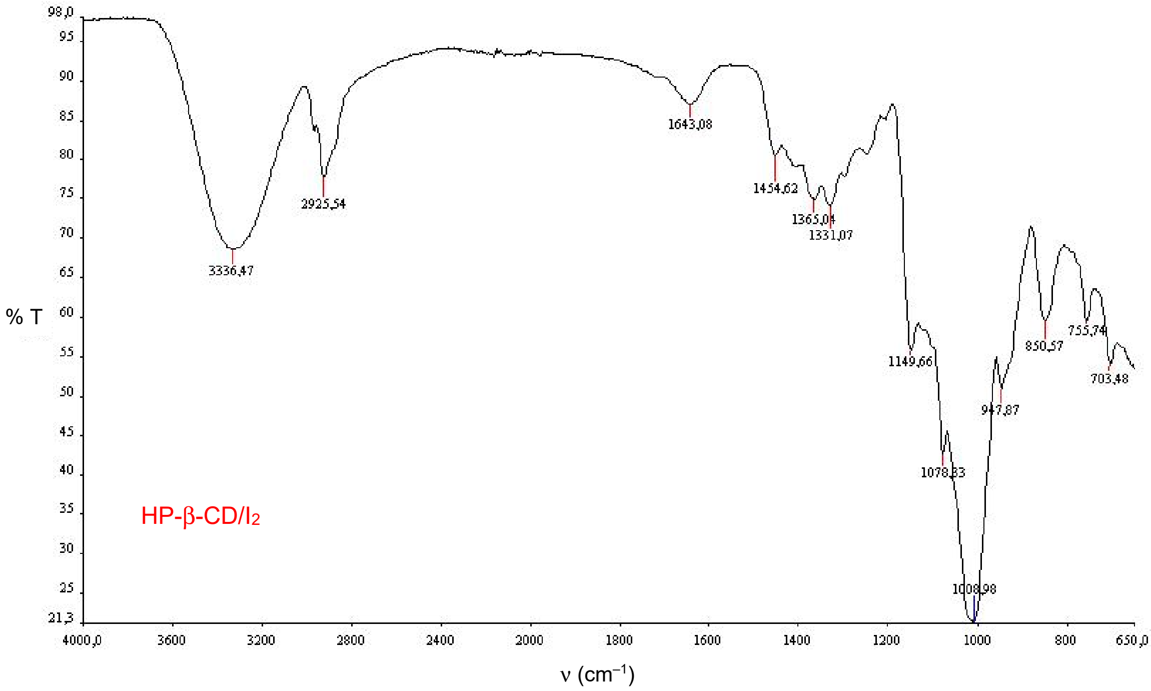

Figure S1. IR spectra of HP-β-CD (top) and HP-β-CD/I_2 complex (down)

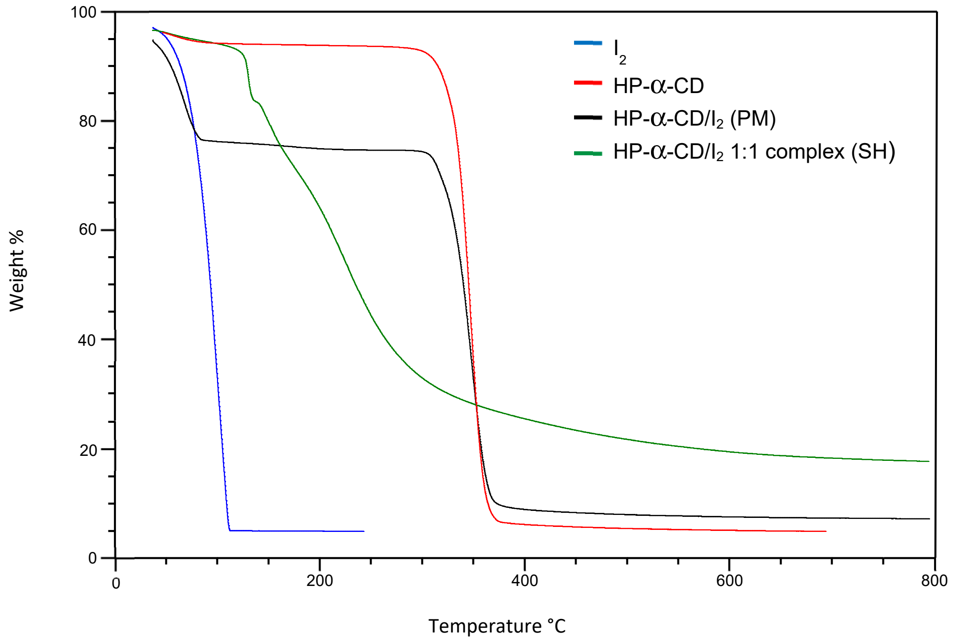

Figure S2. TG curves of iodine and HP-α-CD as pure components, their 1:1 physical mixture (PM) and 1:1 complex prepared by sealed heating (SH) method

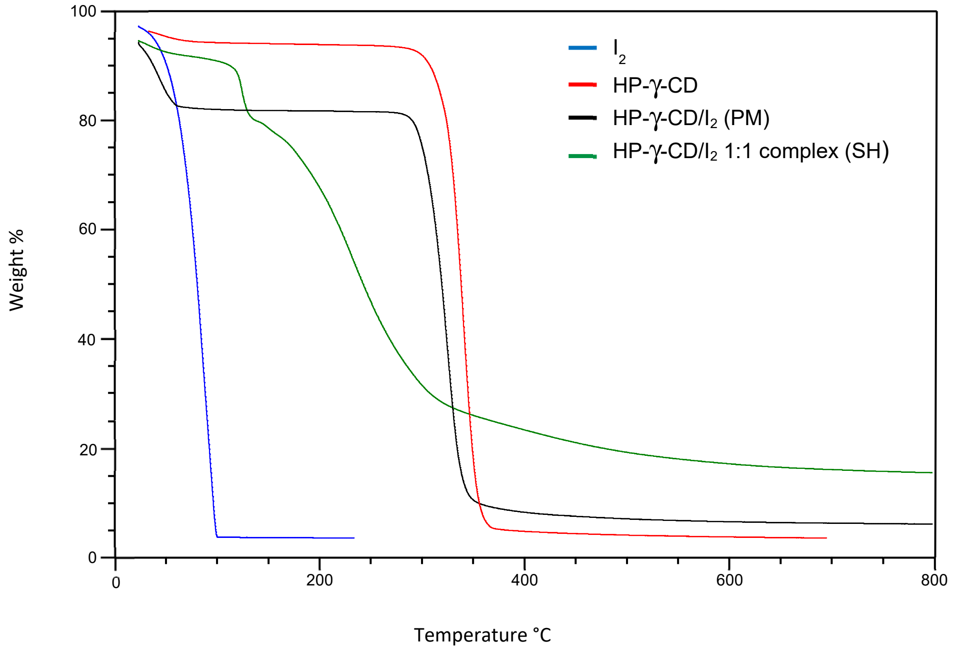

Figure S3. TG curves of iodine and HP-γ-CD as pure components, their 1:1 physical mixture (PM) and 1:1 complex prepared by sealed heating (SH) method

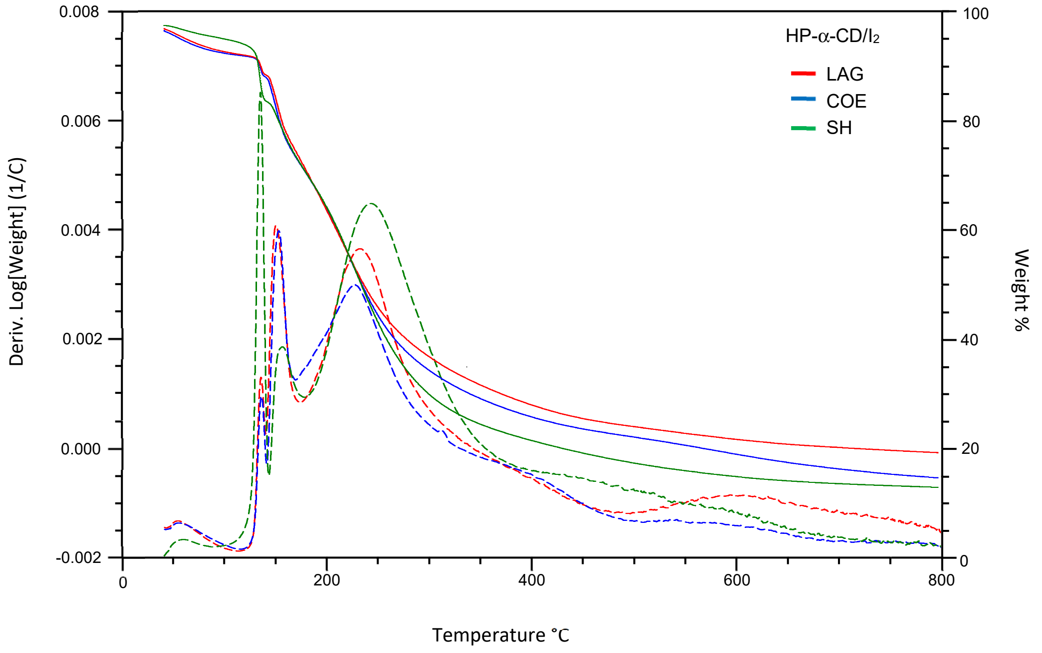

Figure S4. TG (solid lines) and first derivative (dotted lines) curves of HP-α-CD/I_2 complexes prepared by liquid-assisted grinding (LAG), co-evaporation (COE) and sealed heating (SH) methods

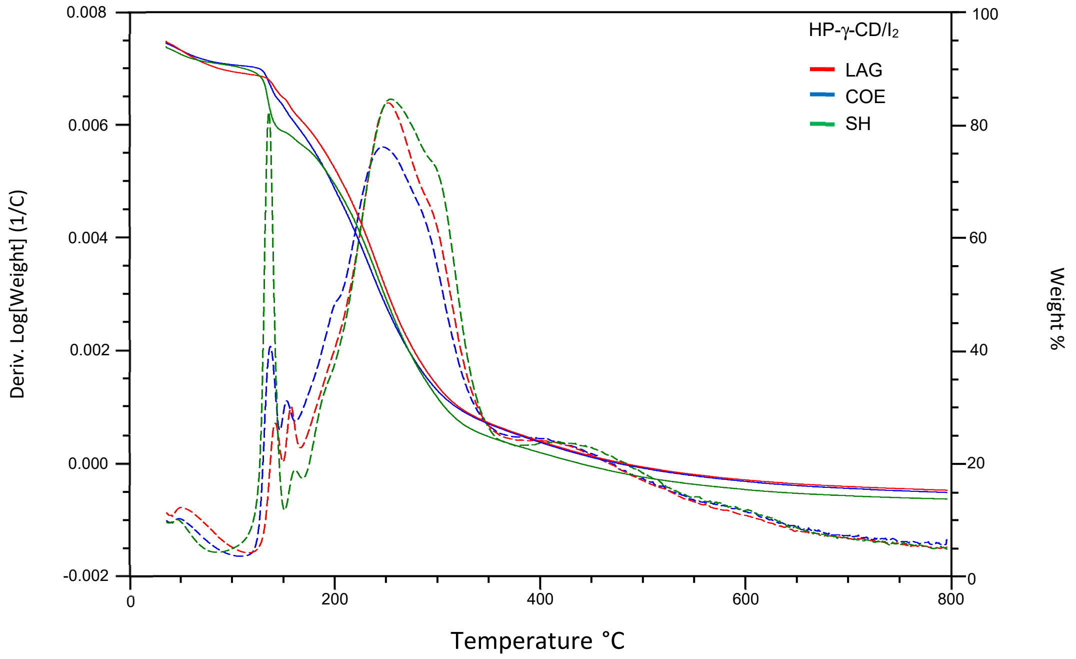

Figure S5. TG (solid lines) and first derivative (dotted lines) curves of HP-γ-CD/I_2 complexes prepared by liquid-assisted grinding (LAG), co-vaporation (COE) and sealed heating (SH) methods.

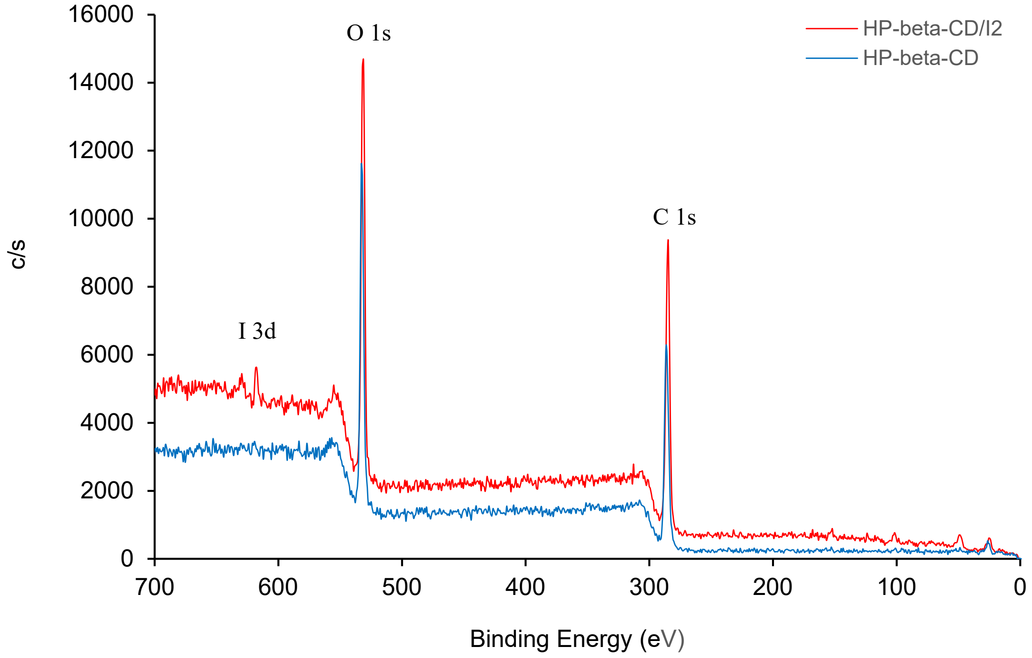

Figure S6. XPS survey spectrum of HP-β-CD and HP-β-CD/I_2 (SH sample) solids

Table S2. Accelerated stability test on solid iodine-cyclodextrin complexes[a]

Cyclodextrin	Preparation method	% I_2 (w/w)							
		t = 0	*t = 1*[b]	*t = 2*[b]	*t = 6*[b]	*t = 7*[b]	*t = 14*[b]	*t = 21*[b]	*t = 28*[b]
HP-α-CD	SH	10.81±0.01	10.29±0.05	10.02±0.01	9.73±0.05	9.56±0.06	9.20±0.04	8.59±0.02	8.09±0.01
HP-α-CD	LAG	10.60±0.08	10.10±0.03	9.66±0.05	9.21±0.06	9.14±0.03	8.86±0.02	8.83±0.03	8.61±0.01
HP-α-CD	COE	9.45±0.03	9.19±0.02	9.03±0.04	8.82±0.03	8.80±0.06	8.82±0.04	8.74±0.04	8.73±0.02
HP-β-CD	SH	9.64±0.03	8.98±0.05	8.46±0.02	7.74±0.03	7.43±0.08	6.98±0.06	6.79±0.04	6.40±0.01
HP-β-CD	LAG	5.14±0.05	4.92±0.03	4.70±0.04	4.38±0.03	4.31±0.06	4.16±0.04	4.03±0.03	3.69±0.01
HP-β-CD	COE	6.01±0.04	5.92±0.03	5.81±0.05	5.56±0.04	5.58±0.04	5.47±0.03	5.53±0.06	5.36±0.02
HP-γ-CD	SH	8.32±0.11	7.10±0.07	6.49±0.05	4.60±0.07	4.28±0.07	3.94±0.05	3.54±0.06	3.22±0.01
HP-γ-CD	LAG	5.56±0.03	5.18±0.01	4.91±0.02	4.63±0.01	4.46±0.01	4.50±0.02	4.42±0.03	4.39±0.05
HP-γ-CD	COE	6.77±0.06	6.48±0.07	6.23±0.05	5.57±0.04	5.50±0.05	4.99±0.06	4.76±0.04	4.57±0.03

[a]Each solid iodine-cyclodextrin complex was spread in a glass Petri dish (40 mm diameter) and stored at 40 °C in oven; [b]Time in days

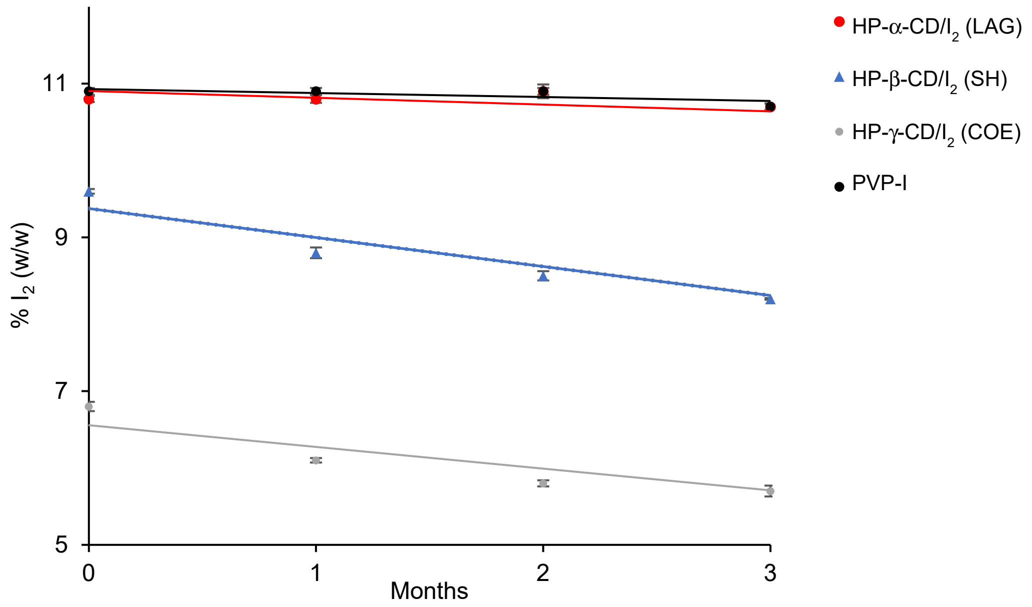

Figure S7. Variation of the iodine content in solid complexes with 2-hydroxypropylcyclodextrins on storage at 25 °C in low-density polyethylene bag

Table S3.Variation of iodine content of iodine-cyclodextrin complexes in solution[a]

Cyclodextrin	Preparation method	% I_2 (w/w)			
		t = 0	*t = 1*[b]	*t = 2*[b]	*t = 3*[b]
HP-α-CD	SH	10.78±0.04	8.10±0.03	6.78±0.04	5.97±0.08
HP-α-CD	LAG	10.61±0.09	6.63±0.09	5.59±0.07	4.72±0.02
HP-α-CD	COE	9.42±0.08	6.16±0.02	5.10±0.06	4.25±0.04
HP-β-CD	SH	9.59±0.05	1.62±0.06	0.64±0.01	0
HP-β-CD	LAG	5.13±0.05	1.24±0.06	0	0
HP-β-CD	COE	6.02±0.07	1.01±0.04	0	0
HP-γ-CD	SH	8.31±0.02	0	0	0
HP-γ-CD	LAG	5.64±0.07	0	0	0
PVP-I[c]		10.89±0.07	4.84±0.01	0	0

[a]The iodine-cyclodextrin complex was dissolved in water at 0.25% (w/w) concentration and the solution was maintained at 25 °C; [b]Time in months; [c]PVI-I: povidone iodine taken as reference

Table S4. % Inhibition of *S. epidermis* cell viability[a]

Compound	Time								
	10s	*20s*	*40s*	*1 min*	*2min*	*4 min*	*8 min*	*1h*	*6h*
HP-α-CD	100.00	100.00	100.00	100.00	100.00	100.00	100.00	100.00	100.00
HP-β-CD	100.00	100.00	100.00	100.00	100.00	100.00	100.00	100.00	100.00
HP-γ-CD	100.00	100.00	100.00	100.00	100.00	100.00	100.00	100.00	100.00
PVP-I	100.00	100.00	100.00	100.00	100.00	100.00	100.00	100.00	100.00
Vehicle+Penicillin (2μg/mL)	59.27	65.58	71.28	60.50	79.16	72.10	63.42	75.52	95.40
Vehicle (PBS)	56.57	61.54	58.89	73.21	49.62	65.50	66.71	57.78	61.38

[a]Solutions of cyclodextrin-iodine complexes (SH samples) in 0.025% available iodine concentration were used
